# Wearable Activity Trackers Objectively Measure Incidental Physical Activity in Older Adults Undergoing Aortic Valve Replacement

**DOI:** 10.3390/s23063347

**Published:** 2023-03-22

**Authors:** Nicola Straiton, Matthew Hollings, Janice Gullick, Robyn Gallagher

**Affiliations:** School of Nursing and Sydney School of Health Sciences, Faculty of Medicine and Health, University of Sydney, Camperdown, NSW 2006, Australia

**Keywords:** wearable, older adults, aortic valve replacement, physical activity, activity tracker, acceptability, feasibility

## Abstract

Background: For older adults with severe aortic stenosis (AS) undergoing aortic valve replacement (AVR), recovery of physical function is important, yet few studies objectively measure it in real-world environments. This exploratory study explored the acceptability and feasibility of using wearable trackers to measure incidental physical activity (PA) in AS patients before and after AVR. Methods: Fifteen adults with severe AS wore an activity tracker at baseline, and ten at one month follow-up. Functional capacity (six-minute walk test, 6MWT) and HRQoL (SF 12) were also assessed. Results: At baseline, AS participants (*n* = 15, 53.3% female, mean age 82.3 ± 7.0 years) wore the tracker for four consecutive days more than 85% of the total prescribed time, this improved at follow-up. Before AVR, participants demonstrated a wide range of incidental PA (step count median 3437 per day), and functional capacity (6MWT median 272 m). Post-AVR, participants with the lowest incidental PA, functional capacity, and HRQoL at baseline had the greatest improvements within each measure; however, improvements in one measure did not translate to improvements in another. Conclusion: The majority of older AS participants wore the activity trackers for the required time period before and after AVR, and the data attained were useful for understanding AS patients’ physical function.

## 1. Introduction

In patients with aortic stenosis (AS), a change in physical function can act as a marker of disease progression, provide an understanding of overall functional capacity and quality of life, and help determine whether the treatment approaches provided are effective [1]. Yet to date, other than patient self-reports and clinic-based measures of physical function, little real-world objective evidence of actual physical activity exists.

AS is the most common heart valve disease in developed countries. People living with AS often experience symptoms of breathlessness, pain, and fatigue as their condition worsens [2,3], leading to a reduced functional capacity, less engagement in incidental physical activity, such as walking around their home or community, and a poorer health-related quality of life (HRQoL) [4,5]. Such issues are often compounded by aging, multiple comorbidities, and frailty [6]. Aortic valve replacement (AVR), the recommended treatment for severe AS, is performed either through traditional surgical aortic valve replacement (SAVR) or minimally invasive transcatheter aortic valve replacement (TAVR). AVR often leads to improved prognosis, reduced symptoms, and, for most, a restoration of physical function [7].

A change in a person’s physical function can be examined in different ways, such as measuring their functional capacity (i.e., their ability to perform daily activities that require physical exertion) or investigating their engagement in incidental PA. Incidental PA is part of everyday living and includes active transportation (e.g., walking), domestic chores, and non-specific ambulation in domestic settings [8,9]. Declining incidental PA and functional capacity are evident when people with AS experience difficulty or dependency in carrying out activities that are central to independent living, such as self-care tasks, tasks related to living autonomously in one’s own home or role-related activities [10,11]. 

Whilst physical function is important to measure, there are limitations in traditional assessment tools. For instance, the gold standard six-minute walk test (6MWT) often used to examine exercise capacity in older AS patients, is performed only at a single time point and in a clinical setting [12]. Furthermore, self-report questionnaires have been critiqued for their tendency to be age- or disease-biased, often excluding common elements of regular incidental PA performed by older adults (e.g., personal care or domestic tasks). Research-grade motion sensors, such as wearables, can overcome some of these issues by directly tracking the activity of individuals in their everyday surroundings as they go about their usual activities. The importance of monitoring physical function in AS patients before and soon after AVR is essential for providing appropriate care and support for more frail or deconditioned individuals, optimizing health outcomes, and identifying cardiac rehabilitation opportunities [13].

The evidence supporting the accuracy and validation of wearables to monitor activity in people with a range of different health conditions is growing. In a study of adults with congenital heart disease (ACHD), wearables were used to compare objectively measured and self-reported PA with a healthy cohort, finding that people with ACHD walked, on average, 8000 steps daily but had lower activity intensity [14]. Likewise, for cardiac patients (average age 65 years) participating in a rehabilitation program, wearables were found to be a useful tool for assessing the attainment of PA guideline recommendations yet challenges with overestimation of step count and activity levels were reported [15]. Our systematic review, examining a range of wearables, also validated the use of trackers to measure step count and activity levels amongst older adults (average age 70 years) in community-dwelling settings [16]. However, studies in this review also reported practical challenges around the usability of wearables by older adults, including unfamiliarity with the technology itself and concerns about data provision, all potential key barriers impacting future adoption.

Despite the rising validation evidence base for the use of wearables to monitor PA and identification of data acquisition challenges, little evidence exists regarding the actual utility of such devices amongst older, frail adults, such as those living with AS. Therefore, this exploratory study aimed to examine the acceptability and feasibility of wearable trackers to measure real-world incidental PA in older adults living with AS before and after AVR. Incidental PA will also be measured alongside exercise capacity (6MWT) and generic HRQoL (SF12) to determine whether changes corresponded. The evidence attained may act as a steppingstone to inform the consideration and use of wearable trackers amongst older adults in future studies.

## 2. Materials and Methods

### 2.1. Study Design

This exploratory study examined the acceptability and feasibility of monitoring incidental PA in patients with AS before and one month after AVR. Incidental PA was measured in free-living environments using a wrist-worn, wearable activity tracker for four consecutive days and was assessed alongside exercise capacity (6MWT) and generic HRQoL (SF12). This study was part of a larger observational study exploring functional capacity, PA, social support, and HRQoL in people living with AS before and after AVR.

### 2.2. Subjects and Setting

A consecutive recruitment approach was used, enrolling AS patients from three tertiary hospital sites in Sydney, Australia, between 2016 and 2020. Consecutive recruitment was used to avoid bias and ensure maximum enrolment due to the limited TAVR procedural numbers across all sites at the time this study was conducted. 

Participants were eligible if they: (1) were 75 years or older; (2) had a diagnosis of severe AS diagnosis (defined as peak transvalvular gradient of ≥40 mmHg on transthoracic echocardiography or transesophageal echocardiography or an aortic valve area of <1.0 cm^2^); (3) were recommended to undergo AVR, either SAVR or TAVR; (4) had heart failure symptoms classified as New York Heart Association (NYHA) Functional Class ≥ II before AVR; (5) could understand the English language sufficient to provide informed consent and undertake the questionnaires; and (6) could wear an activity tracker (Fitbit Flex) for four consecutive days to measure incidental PA. 

The age range inclusion criteria were chosen in alignment with guideline treatment recommendations for severe AS at the time this study was conducted. In Australia, the guidelines for older patients with AS undergoing AVR do not specify that they should be referred to formal cardiac rehabilitation. However, individual healthcare providers can refer on the basis of the patient’s specific needs and suitability.

### 2.3. Data Collection and Measures

Incidental PA, exercise capacity, and HRQoL assessments were performed before AVR (baseline) and after AVR at follow-up (1 month), in alignment with routine clinic visits. The acceptability and feasibility of wearable activity trackers were assessed by examining factors, such as participant compliance in wearing the device for the specified monitoring period, participant retention, and the adequacy of the data collected for analysis.

#### 2.3.1. Demographic and Clinical Data

Clinical and sociodemographic data were recorded at baseline and summarized the AS participant characteristics, including age, sex, marital status, comorbidities (Charlson Comorbidity Index), New York Heart Association (NYHA) functional class, and surgical-risk score (STS). The KATZ index of independence was used to assess function as a measurement of the person’s ability to perform activities of daily living independently.

#### 2.3.2. Physical Activity and Functional Capacity

Objective incidental PA and functional capacity were assessed using two repeated measures: step count and 6MWT. 

The feasibility of using trackers to record physical activity in the population was evaluated by assessing the trackers’ capacity to capture incidental physical activity through daily step counts. Wearables have demonstrated accuracy in recording step count in other populations [17,18]. We captured step count at baseline and one month post-AVR. To evaluate the acceptability of the trackers, we gathered data on the number of days participants wore them throughout the study duration. 

A wrist-worn, wearable activity tracker, such as the Fitbit-Flex (Fitbit Inc., San Francisco, CA, USA) used in this study, is a small and light accelerometer used to capture average steps taken per day [19]. The Fitbit Flex uses a three-dimensional AC accelerometer to sense user movement to calculate steps walked. A higher number of steps taken indicates higher engagement in incidental PA. 

PA is defined as any movement that increases energy expenditure beyond the resting state. It can be classified into three categories on the basis of the intensity of effort required to perform the activity: light (low energy expenditure, such as walking at a slow pace), moderate (moderate energy expenditure, such as brisk walking) and vigorous (high energy expenditure, such as jogging). The Compendium of Physical Activities provides a comprehensive list of PA and their associated metabolic equivalent of the task (MET) values [20]. The Compendium quantifies the types of PA described in this study; for instance, light intensity is classified as 1.6–2.9 METs, moderate intensity 3–5.9 METs, and vigorous intensity ≥6 METs. However, it is widely acknowledged that wearables, such as the Fitbit utilized in this study, use proprietary algorithms to determine activity and energy expenditure thresholds [21]. Weighted moderate to vigorous physical activity (MVPA) is an established way of measuring how much physical activity people engage in during a day and is often used to assess health risks and benefits from physical activity [22].

The 6MWT assesses functional exercise capacity by capturing distance (metres) walked over six minutes. The test was clinician-administered and performed by participants on a straight and unobstructed 30-metre track in the clinic environment at each site. Data were measured and recorded using a trundle wheel and stopwatch to capture the total distance walked in metres over the six minutes. Higher distance walked is better, and the minimal clinically important difference when comparing pre- and post-measurements often includes improvements of ≥50 m [23]. 

Additional baseline functional capacity measures included the five-metre walk test (5 mWT) to examine gait speed (seconds) and an individual’s functional mobility and a grip strength assessment using a handheld dynamometer (Jamar©, Bolingbrook, IL, USA) [24,25]. 

#### 2.3.3. Health-Related Quality of Life

The Medical Outcomes Study Short-Form 12 (SF-12) Health Survey was used to assess health-related quality of life. It is a 12-item self-administered questionnaire used to capture generic health outcomes from the patient’s perspective [26]. The SF-12 measures eight health domains (on a Likert scale): physical functioning (PF), role-physical (RP), bodily pain (BP), general health (GH), vitality (VT), social functioning (SF), role-emotional (RE), and mental health (MH), which are summarized into a Physical Health Component Score (PCS) and a Mental Health Component Score (MCS). Scores > 50 indicate better physical or mental health than the mean, and scores < 50 indicate worse health (higher scores are better). Scores for each domain range from 0 to 100, where higher scores represent better health. This questionnaire has been recommended by the Valve Academic Research Consortium to evaluate HRQoL pre- and post-AVR [27]. 

### 2.4. Procedure

Patients with severe AS scheduled to undergo AVR (SAVR or TAVR) were invited to participate in this study. After eligibility was confirmed, written informed consent was obtained, and the patients clinical and sociodemographic data were extracted from their medical records. Patients were then asked to complete the study questionnaires and, under supervision from the research team, perform the functional capacity assessments, including 6MWT and gait and grip assessments in the clinic environment of the study site. After preparing the Fitbit device, each patient was provided with a brief written guide on device placement, wear time, and how to troubleshoot any problems with the device. Patients were instructed to place the Fitbit-Flex on the non-dominant hand and to wear the device for four consecutive days in their own environments during waking hours (at least 10 h), except when showering. Patients were informed that data collection would be repeated at an assessment 1 month post-AVR. All assessments took place in the same location as the baseline assessment. All patients were blinded to their Fitbit-Flex data throughout the study.

### 2.5. Data Analysis

Data analyses were performed using the Statistical Package for the Social Sciences (SPSS), version 27 (IBM, New York, NY, USA). SPSS was used to calculate mean and median scores, standard deviations (SD), and inter-quartile ranges (IQR), while percentages (%) were used to describe the socio-demographic and clinical characteristics of the participants. The relative change scores (i.e., (post-value − pre-value) ÷ pre-value) for the three measures (step count, 6MWT, and HRQoL) were assessed at baseline and repeated at 1 month. Two-sided paired t-tests were used to further analyze 6MWT and HRQoL, and Pearson’s correlation coefficients were computed to assess the association between clinical assessments, incidental PA outcomes, and key clinical and demographic variables. R studio v1.4.1106 (R Foundation for Statistical Computing, Vienna, Austria) was used to calculate correlations, with visualization using the ‘corrplot’ package [28]. The level of significance was set at *p* < 0.05. 

This was an exploratory analysis on this small dataset to help inform larger studies that may be powered to conduct analyses of change scores.

## 3. Results

**Figure 1:**
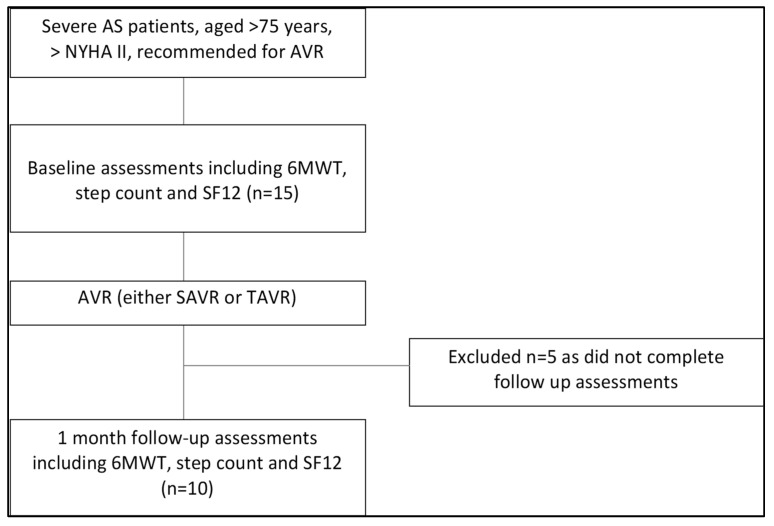
PRISMA diagram.

Fifteen patients with severe AS consented to participate in the study. All were able to perform the incidental PA, functional capacity, and HRQoL measures at baseline and underwent an AVR procedure (see Figure 1). Of the 15 participants who completed the functional capacity, incidental PA, and HRQoL measures at baseline, only ten were able to complete the same measures one month after AVR; the remaining five were unable to complete them as they did not attend their follow-up visit at the study sites.

The demographic and clinical characteristics of the 15 participants at baseline are presented in Table 1. The mean age of participants in this study was 82.3 ± 7.0 years, 53.3% were female, and 46.7% were married. At baseline, left ventricular ejection fraction was predominantly normal (58.3 ± 11.2), yet all expressed symptoms of heart failure as classified by the New York Heart Association Class, 73.3% (III) and 26.7% (II), respectively. Chronic comorbidities were common—66.7% scored 1–2, and 26.6% scored 3–4 on the Charlson Comorbidity Index—and cardiovascular disease was prominent, including ischemic heart disease and peripheral vascular disease. The surgical-risk score (STS) score is commonly used with patients undergoing SAVR but is growing in application amongst the TAVR population. The overall mean STS score for this group was 5.5 ± 4.5%, indicating the sample at baseline to be of intermediate operative risk. 

At baseline, of the total potential 60 days (15 patients × 4 consecutive days each) of tracker wear time for all participants, data were captured for a total of 52 days (Table 2). For the incidental PA that was measured by the Fitbit Flex, a median daily step count of 3437 steps per day, IQR 5337, was recorded. Functional capacity varied, as measured by the 6MWT, with a median 6MWT distance of 272 m (IQR 116.75), ranging from 102 m (slow walker) to 365 m (fast walker). Participants also had a mean gait speed of 0.84 ± 0.15 m per second and a mean grip strength (for both sexes) of 18.57 ± 3.66 kg.

For HRQoL at baseline, measured with the SF-12, mean physical component score (PCS) of 41.8 ± 4.9 and a mean mental component score (MCS) of 48.1 ± 12.6 were recorded. Finally, the majority of participants underwent a TAVR (73.3%) vs. SAVR (26.7%) procedure.

Ten participants completed the follow-up assessments (Table 2). The potential overall wear time for the trackers across all participants was a total of 40 days (10 patients × 4 consecutive days each), yet actual data were captured for a total of 36 days. At one month after AVR, comparing the ten participants with baseline and follow-up data, there was a mean difference reduction of −562.2 ± 1726.6 steps per day compared with baseline but a mean difference improvement of 26.6 ± 76.2 m on 6MWT (*p* = 0.404). The mean difference change in PCS was significant and more (7.69 ± 6.57, *p* = 0.005) than MCS (1.49 ± 7.22, *p* = 0.531) at one month.

**Figure 2:**
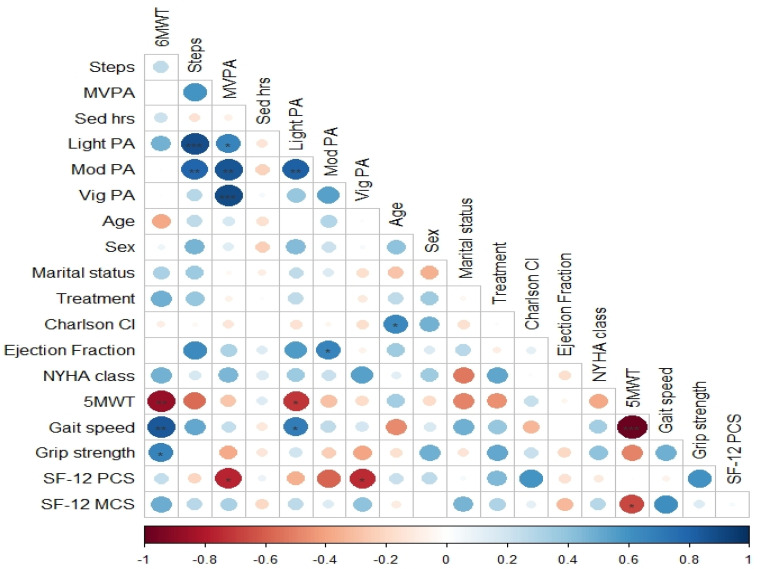
Correlation matrix: association between functional capacity assessments, incidental PA outcomes, and key clinical and demographic variables pre- and post-AVR. 6MWT = six-minute walk test, Steps = average steps per day, MVPA = mean vigorous physical activity, Sed hrs = sedentary hours, Light PA = light physical activity, Mod PA = moderate physical activity, Vig PA = vigorous physical activity, and 5MWT = gait speed.

At one-month post AVR, participants with the lower baseline step count (e.g., lowest 123 steps/day improved to 2031 steps/day), 6MWT (e.g., lowest 102 m improved to 356 m), and PCS (e.g., lowest 31.77 points to 45.83) noticed the greatest improvements. Across these ten participants, the variation in individual change steps per day, distance walked, and HRQoL at one month was wide-ranging. For several participants, an improvement in one measure did not translate to direct improvements in another measure. Three participants had an improved 6MWT at follow-up of 16–27%, alongside a reduced step count incidental PA by 9–65% and a variable change, (0.1–24%) in HRQoL (see Table 3). There were no associations between change in 6MWT and change in objective PA measures (see Figure 2 and Supplementary Table S1).

## 4. Discussion

The purpose of this exploratory study was to examine the acceptability and feasibility of using wearable trackers to measure incidental PA in older, frail adults with severe AS before and after AVR. 

In our study, before AVR, these older AS participants were symptomatic and managing multiple comorbidities. Overall, their left ventricular ejection fraction was in the normal range (mean 58%) and reflected the adoption of emerging treatment guidelines for AS patients undergoing AVR at the time this study was conducted. Participants were of intermediate surgical risk, and most underwent a TAVR procedure. Using common frailty as assessment measures, these AS participants had worse functional capacity, indicated by strength and walking speed, and much lower HRQoL than adults of a comparable age [29,30,31]. This highlights the debilitating impact of untreated severe, symptomatic AS and its impact on people’s physical function.

The acceptability of wearable trackers was evaluated by examining the actual number of days the devices were worn versus the total potential number of days data could have been collected. At baseline and follow-up, incidental PA was captured for 85–90% of the potential total wear time for all participants. Reasons for no data capture days included the tracker not being worn (patient removed due to discomfort or removed and could not put back on), worn but activity not captured, and issues with battery power. These findings align with challenges reported from similar studies investigating the use of wearables amongst older adults, including reports of the devices being uncomfortable, difficult to use (fine motor movements required to attach the device and use the functions), and low visual accessibility of the device features (e.g., readability of display units) [32,33]. It is critical to assess the usability of wearable activity trackers in older adults recognizing that they may have different physical and cognitive abilities, and different life experiences, preferences, and lifestyles than those of younger adults [34]. 

However, as the research involving wearables expands, it is still recognized that there is a lack of structured methodological approaches to guide the design of studies to effectively assess the acceptability and feasibility of wearables. Recent literature suggests a five-step approach could be adopted to examine the usability of wearable systems, including (1) defining the target users; (2) conducting a task analysis for identifying the context, the parameters to be measured, and the methodology to collect data; (3) preparing a protocol and investigation tools; (4) executing usability experiments; and (5) consider different ways to interpret and report the data [35]. In this study, although we considered several of these approaches, we could not implement them all for various reasons, including the exploratory design of the study, participant burden, availability of appropriate usability questionnaires, and limited access to the range of available wearable devices. Data were collected, as per Table 2, regarding how long participants wore the trackers, and ‘reasons for no data captured’ were documented, both useful data points that begin to examine the acceptability of wearables. 

Furthermore, although patients can perform PA on request in a clinic environment (e.g., 6MWT), at the same time point in their home environments, they may actively choose to avoid activity and recover at their own pace. Whilst this is no surprise, few studies report it or examine differences in activity levels (clinic vs. free-living) or, indeed, the impact of these variations on the long-term recovery of physical function [36]. Exploring what this means in the context of achieving expected clinical outcomes post-AVR may be useful to patients, carers, and clinicians and help them to better manage recovery expectations accordingly. This is important to consider, as in Australia, it is not commonplace for older adults, such as those post-AVR, to attend or be referred to cardiac rehabilitation programs despite the known benefits [32]. Wearables in this context, therefore, could act as a valuable monitoring tool pre- and post-AVR, providing useful indications of AS patient’s actual incidental PA in their environments. This information may help them understand their physical well-being and assist practitioners to prescribe more personalized recovery recommendations [37]. 

In previous studies, the Fitbit has proved to be a reliable device for capturing these data among older adults with AS and highlighted that at both pre- and post-AVR, these patients were taking much fewer steps per day compared with similar-aged, community-dwelling adults (3000–3500 steps per day versus a mean of 7000 steps per day respectively) [16]. Our results are, however, similar to those from a recent pilot study monitoring PA in AS patients after TAVR using wearables and found that participants were achieving 3000–4000 steps per day [38]. Both their data and ours could be used to help understand how AS patients function in their home environments before and after AVR and help identify those in need of more support to improve physical activity to ensure a better recovery, quality of life, and prognosis. However, both studies reported an issue with participant retention at follow-up with, on average, a 30–40% drop-out rate. Reasons for this included non-compliance with study activities and participants declining to continue in the study. These findings raise salient points with regard to acceptability and need to be considered early to accurately assess the feasibility of activity trackers amongst more complex populations, such as older adults [39]. 

We also found at one month post-AVR, those participants with poorer baseline measures of incidental PA, functional capacity, and HRQoL improved the most, aligning with findings from similar studies [40,41]. However, our results also highlighted that improved functional capacity post-AVR in the clinic setting does not always correspond to improved engagement in incidental PA in the free-living environment and vice versa. Furthermore, improved physical function did not always translate to better quality of life scores one month after AVR, especially mental well-being. Comorbidities, low mood, and social and geographical isolation are known factors that can impact older adult’s recovery post-cardiac procedures and may explain some of the results observed [42,43]. Similar studies exploring the use of wearables across different patient populations also report disagreements between self-reported PA and objectively measured PA. Whilst this may be expected, our study highlights the lack of agreement between two objective measures of PA. 

The contribution of this paper is that it is one of the few emerging studies measuring incidental activity in a population of older, frail adults, who are commonly not included in such assessments. Using wearables to do this may help both clinicians and AS patients understand their cardiovascular fitness and help identify opportunities to reverse any physical deconditioning either before or after AVR [44]. This is an important issue for consideration given the increase in the aging population, and the expected growth in the number of people being diagnosed with AS in the coming years [45]. Furthermore, learnings arising from this study in terms of the data attained, challenges experienced, and study design implemented, provide evidence that may help guide future research approaches using wearable trackers to measure PA in older adults.

## 5. Limitations

While the methods utilized were acceptable for this exploratory study, further research should be adequately powered to determine statistical significance as a basis for research and practice decisions. More standardized measures to assess the usability of wearables would have also benefited the evaluation of the data attained. However, a study strength was the use of multiple validated methods to explore and measure changes in real-world PA, functional capacity, and HRQoL of patients with AS, before and after AVR. 

## 6. Conclusions

For the majority of older AS patients, wearing the activity trackers during the necessary time period, both before and after AVR, was both acceptable and feasible. The data collected through these trackers provided valuable information regarding AS patients’ physical function in real-world settings and might help identify and support these individuals pre- and post-interventions. Future research should also consider the effectiveness of measuring incidental PA on a larger scale using wearable trackers and assess the utility of these devices in the management of older adults before and after valve replacement.

## Figures and Tables

**Table 1:** Baseline participant demographic and clinical characteristics.

(*n* = 15)	
**Socio-demographics**	
Age, years, mean ± SD	82.3 ± 7.0
Female, %	53.3%
Married, %	46.7%
**Clinical characteristics**	
Left ventricular ejection fraction, % mean ± SD	58.3 ± 11.2
New York Heart Association class, n (%)	
II	4 (26.7%)
III	11 (73.3%)
Society of Thoracic Surgeons Score, %, mean ± SD	5.5 ± 4.5
**Charlson Comorbidity (CCI) Index**	
CCI 1–2 (mild), %	66.7%
CCI 3–4 (moderate), %	26.6%
CCI >5 (severe), %	6.7%
**Functional capacity**	
Katz Index (activities of daily living), mean ± SD	6.00 ± 0.0
6 min walk test (6MWT), total metres, median (IQR)	272 (116.75)
Grip strength, kilograms, mean ± SD	18.57 ± 3.66
Gait speed metres per sec, mean ± SD	0.84 ± 0.15
**Physical activity**	
Steps per day, median (IQR)	3437 (5336.81)
**SF-12**	
Physical component score (PCS), mean ± SD	41.8 ± 4.9
Mental component score (MCS), mean ± SD	48.1 ± 12.6
**Procedures**	
Transcatheter aortic valve replacement, n (%)	11 (73.3%)
Surgical aortic valve replacement, n (%)	4 (26.7%)

Society of Thoracic Surgeons mortality risk model (≥8% = high risk; 4–7% intermediate risk; <4% = low risk), Charlson Comorbidity Index (1–2% = mild, >5% = severe) and Katz Index of Independence in Activities of Daily Living (6 = full function; ≤2 = severe functional impairment).

**Table 2:** Total tracker data capture across all participants before and after AVR.

Timepoint (No. of Participants)	Total vs. Actual Data Capture (Days)	Reasons for No Data Captured
Baseline (*n* = 15)	60 vs. 52	One participant wore device but no data capture 3 of 4 days; two participants removed device after 48 h due to discomfort
Follow-up (*n* = 10)	40 vs. 36	One participant removed device after 72 h and could not reapply to wrist; one participant wore device but no data capture 1 of 4 days; one participant wore device, but battery stopped working on Day 4

**Table 3:** Functional capacity, incidental physical activity, and HRQoL before and after AVR.

Participant	Age	Baseline				1 Month							
		6MWT (m) *	STEPS/DAY **	PCS ^+^	MCS ^++^	6MWT (m)	6MWT, % Change	STEPS/DAY	Step Count, % Change	PCS	PCS, % Change	MCS	MCS, % Change
10	76	102	123	42.23	41.17	356	249	2031	1558	54.21	28	56.03	36
23	83	165	4627	36.41	56.26	201	22	4215	−9	51.16	41	56.43	0.1
3	88	196	742	47.66	26.82	210	7	1782	140	50.91	7	23.00	−14
20	79	219	8774	43.71	59.73	253	16	6286	−28	42.48	−3	59.27	−1
13	99	268	602	38.82	55.61	268	0	773	28	53.50	38	56.87	2
14	80	276	3534	44.24	38.41	202	−27	4008	13	41.85	−5	42.32	10
6	75	296	7359	47.64	48.06	377	27	2560	−65	59.16	24	50.70	5
1	81	326	7120	31.77	66.51	245	−25	3623	−49	45.83	44	57.91	−13
2	86	336	2413	43.05	50.40	290	−14	2290	−5	49.20	14	61.39	22
15	79	365	3341	44.54	63.30	413	13	5446	63	48.64	9	57.23	−10

* 6MWT, six-minute walk test, measured in metres walked, higher is better; ** Average steps per day, measured by the Fitbit wearable tracker over four consecutive days; ^+^ SF12 PCS, physical component score, range of 0–100, higher is better; ^++^ SF12 MCS, mental component score, range of 0–100, higher is better. Colour coding: green = improved, yellow = no change, and red = declined.

## Data Availability

The data presented in this study may be available on request from the corresponding author. The data are not publicly available due to privacy.

## Supplementary Materials

The following supporting information can be downloaded at: https://www.mdpi.com/article/10.3390/s23063347/s1, Table S1: change correlation matrix.
