# Supplementary material for: Wearable Activity Trackers Objectively Measure Incidental Physical Activity in Older Adults Undergoing Aortic Valve Replacement
Source: Sensors (Basel). 2023 Mar 22;23(6):3347. doi: 10.3390/s23063347 (PMC10051559; doi:10.3390/s23063347)
Supplement: Supplementary file 1 [file sensors-23-03347-s001.zip › Supplementary Table S1_change correlation matrix_22032022_clean.pdf]

Supplementary Table S1: Change correlation matrix

As per Figure 2, and the values below, there were no associations between change in 6MWT and change in objective PA measures.

| | 6MWT | Steps | MVPA | Sed hrs | Light PA | Mod PA | Vig PA | Age | Sex | Marital status | Treatment | Charlson CI | Ejection Fraction | NYHA class | 5MWT | Gait speed | Grip strength | SF-12 PCS | SF-12 MCS |
|---|---|---|---|---|---|---|---|---|---|---|---|---|---|---|---|---|---|---|---|
| **6MWT** | 1 | | | | | | | | | | | | | | | | | | |
| **Steps** | 0.258 | 1 | | | | | | | | | | | | | | | | | |
| **MVPA** | -0.001 | 0.595 | 1 | | | | | | | | | | | | | | | | |
| **Sed hrs** | 0.213 | -0.151 | -0.088 | 1 | | | | | | | | | | | | | | | |
| **Light PA** | 0.471 | 0.890*** | 0.663* | -0.142 | 1 | | | | | | | | | | | | | | |
| **Mod PA** | 0.015 | 0.796** | 0.858** | -0.222 | 0.814** | 1 | | | | | | | | | | | | | |
| **Vig PA** | -0.015 | 0.287 | 0.896*** | 0.048 | 0.382 | 0.541 | 1 | | | | | | | | | | | | |
| **Age** | -0.383 | 0.256 | 0.171 | -0.161 | -0.008 | 0.299 | 0.022 | 1 | | | | | | | | | | | |
| **Sex** | 0.066 | 0.464 | 0.138 | -0.245 | 0.432 | 0.22 | 0.037 | 0.409 | 1 | | | | | | | | | | |
| **Marital status** | 0.326 | 0.365 | -0.03 | -0.092 | 0.251 | 0.15 | -0.179 | -0.287 | -0.356 | 1 | | | | | | | | | |
| **Treatment** | 0.481 | 0.381 | -0.072 | 0.021 | 0.254 | -0.008 | -0.111 | 0.255 | 0.356 | -0.048 | 1 | | | | | | | | |
| **Charlson CI** | -0.091 | -0.032 | -0.133 | -0.002 | -0.159 | -0.053 | -0.173 | 0.648* | 0.47 | -0.168 | 0.028 | 1 | | | | | | | |
| **Ejection Fraction** | 0.006 | 0.631 | 0.312 | 0.142 | 0.57 | 0.664* | -0.062 | 0.354 | 0.15 | 0.271 | -0.099 | 0.115 | 1 | | | | | | |
| **NYHA class** | 0.476 | 0.174 | 0.451 | 0.159 | 0.369 | 0.223 | 0.546 | 0.124 | 0.356 | -0.524 | 0.524 | 0.028 | -0.176 | 1 | | | | | |
| **5MWT** | -0.860** | -0.562 | -0.274 | 0.148 | -0.711* | -0.3 | -0.189 | 0.346 | -0.189 | -0.492 | -0.451 | 0.231 | -0.057 | -0.384 | 1 | | | | |
| **Gait speed** | 0.840** | 0.519 | 0.247 | -0.13 | 0.694* | 0.254 | 0.186 | -0.476 | 0.163 | 0.481 | 0.389 | -0.33 | 0.005 | 0.344 | -0.987*** | 1 | | | |
| **Grip strength** | 0.662* | 0.002 | -0.373 | -0.135 | 0.197 | -0.249 | -0.396 | -0.161 | 0.484 | -0.146 | 0.511 | 0.221 | -0.21 | 0.402 | -0.492 | 0.486 | 1 | | |
| **SF-12 PCS** | 0.249 | -0.211 | -0.764* | 0.088 | -0.352 | -0.582 | -0.748* | 0.221 | 0.266 | 0.044 | 0.442 | 0.593 | -0.154 | -0.117 | 0.014 | -0.066 | 0.603 | 1 | |
| **SF-12 MCS** | 0.498 | 0.28 | 0.328 | -0.202 | 0.264 | 0.149 | 0.408 | -0.107 | 0.002 | 0.468 | 0.301 | 0.112 | -0.325 | 0.281 | -0.663* | 0.616 | 0.148 | 0.032 | 1 |
